# Effect of Elevated CO_2_ Concentration, Elevated Temperature and No Nitrogen Fertilization on Methanogenic Archaeal and Methane-Oxidizing Bacterial Community Structures in Paddy Soil

**DOI:** 10.1264/jsme2.ME16066

**Published:** 2016-09-07

**Authors:** Dongyan Liu, Kanako Tago, Masahito Hayatsu, Takeshi Tokida, Hidemitsu Sakai, Hirofumi Nakamura, Yasuhiro Usui, Toshihiro Hasegawa, Susumu Asakawa

**Affiliations:** 1 Soil Biology and Chemistry, Graduate School of Bioagricultural Sciences, Nagoya UniversityChikusa, Nagoya, Aichi 464–8601Japan; 2 National Institute for Agro-Environmental Sciences3–1–3 Kannondai, Tsukuba, Ibaraki 305–8604Japan; 3 Taiyokeiki Co., Ltd.Kita-ku, Tokyo 114–0032Japan; 4 Hokkaido Agricultural Research Center, NAROShinseiminami 9–4, Memuro, Kasai, Hokkaido 082–0081Japan

**Keywords:** free-air CO_2_ enrichment (FACE), methanogenic archaea, methane-oxidizing bacteria, paddy field, real-time qPCR

## Abstract

Elevated concentrations of atmospheric CO_2_ ([CO_2_]) enhance the production and emission of methane in paddy fields. In the present study, the effects of elevated [CO_2_], elevated temperature (ET), and no nitrogen fertilization (LN) on methanogenic archaeal and methane-oxidizing bacterial community structures in a free-air CO_2_ enrichment (FACE) experimental paddy field were investigated by PCR-DGGE and real-time quantitative PCR. Soil samples were collected from the upper and lower soil layers at the rice panicle initiation (PI) and mid-ripening (MR) stages. The composition of the methanogenic archaeal community in the upper and lower soil layers was not markedly affected by the elevated [CO_2_], ET, or LN condition. The abundance of the methanogenic archaeal community in the upper and lower soil layers was also not affected by elevated [CO_2_] or ET, but was significantly increased at the rice PI stage and significantly decreased by LN in the lower soil layer. In contrast, the composition of the methane-oxidizing bacterial community was affected by rice-growing stages in the upper soil layer. The abundance of methane-oxidizing bacteria was significantly decreased by elevated [CO_2_] and LN in both soil layers at the rice MR stage and by ET in the upper soil layer. The ratio of *mcrA*/*pmoA* genes correlated with methane emission from ambient and FACE paddy plots at the PI stage. These results indicate that the decrease observed in the abundance of methane-oxidizing bacteria was related to increased methane emission from the paddy field under the elevated [CO_2_], ET, and LN conditions.

Human activities have increased the concentration of atmospheric CO_2_ ([CO_2_]) from approximately 280 to 400 μmol mol^−1^ since pre-industrial times. This concentration is projected to increase further to 470–570 μmol mol^−1^ and, in combination with elevated [CO_2_], global warming (1.0 to 2.0°C) has been anticipated to occur between 2046 and 2065 (17). While these changes will have significant impacts on crop production, they are also expected to affect the rates and/or magnitudes of carbon metabolism and its cycle in the agricultural ecosystem. In lowland rice paddy fields, soil is submerged and represents an important source of methane emission.

Ample evidence exists to show that the projected climate change will enhance methane emission from wet ecosystems (37, 42), which, in turn, will accelerate global climate change. Open-field experiments using a free-air CO_2_ enrichment (FACE) facility have demonstrated that elevated [CO_2_] markedly increases methane emission (16), which is further accelerated by increases in N levels (44) and elevated soil temperatures (33). A mechanism that has been suggested for this enhancement is elevated [CO_2_] enhancing photosynthesis, tillering, and biomass production by rice plants (11, 16, 35), while also stimulating root growth and the supply of carbon to soil. Soil microbial biomass C also significantly increases not only in the rhizosphere (4), but also in bulk soil (15) under elevated [CO_2_]. Elevated temperatures may accelerate carbon metabolism in soil and increase the supply of organic substrates through accelerated root senescence (33). The additional supply of carbon as root biomass and exudates from rice roots serves as a substrate for methanogenesis; therefore, methane production is promoted (4, 16, 24, 34). The addition of nitrogen may stimulate methane emission by enhancing crop growth and/or reducing the C/N ratio (44). These environmental changes in soil have significant impacts on its microbial community, which, in turn, may mediate these processes.

In FACE experiments, the community compositions of methanogenic archaea (12, 23) and methane-oxidizing bacteria (30) were found to be affected by elevated [CO_2_] and elevated soil temperature or low nitrogen fertilization in rice roots and rhizosphere soil. Meanwhile, the abundance of methanogenic archaea and methane-oxidizing bacteria was found to be significantly increased (16, 23, 31) and decreased (31), respectively. On the other hand, although bulk soil markedly contributes to methanogenic and methane-oxidizing processes because it accounts for most of the soil mass, limited information is currently available on the responses of microbial community structures in bulk soil; however, current findings are inconsistent, with the abundance of methanogenic archaea being reported to increase (16, 41) and remaining unchanged (23) with elevations in [CO_2_].

These findings have prompted investigations to clarify whether methanogenic archaea and methane-oxidizing bacteria are influenced in the bulk soil of paddy fields by elevated [CO_2_] and elevated soil temperature or low nitrogen fertilization treatments under FACE conditions. In paddy fields, methane is produced by methanogenic archaea in the reduced layer of plow soil and oxidized by methane-oxidizing bacteria in oxic sites such as the soil surface layer and rhizosphere. The amount of methane emitted from paddy field represents a balance between methane production and oxidation (8), which indicates that the effects of environmental changes such as elevated [CO_2_] and elevated soil temperature on methanogenic archaea and methane-oxidizing bacteria need to be simultaneously evaluated with a focus on the specific soil layers/sites in which the microorganisms function. Recent studies have shown that the simultaneous study of methanogenic archaea and methane-oxidizing bacteria is important for understanding methane metabolism in paddy field soil (6, 8, 10, 20).

Therefore, the present study aimed to elucidate the effects of elevated [CO_2_] (ambient+200 μmol mol^−1^), soil temperature (+2°C), and no nitrogen fertilization on methanogenic archaeal and methane-oxidizing bacterial community structures in bulk soil at different soil depths (0–1 and 1–10 cm) in a FACE experimental paddy field using polymerase chain reaction (PCR)-denaturing gradient gel electrophoresis (DGGE) and real-time quantitative PCR (qPCR).

## Materials and Methods

### Sampling fields

Experiments were conducted at the Tsukuba FACE facility of the National Institute for Agro-Environmental Science (NIAES) in Tsukubamirai city, Ibaraki, Japan (35°58′ N, 139°59′ E) in 2011. Details on the climate and soil properties have already been described by Nakamura *et al.* (28) and Hasegawa *et al.* (11). Briefly, the climate is humid subtropical, with an average annual temperature of 13.8°C and annual precipitation of 1,280 mm. The soil is Fluvisol, containing 21.4 mg g^−1^ total C and 1.97 mg g^−1^ total N. Bulk density was 0.87 Mg m^−3^, with sand, silt, and clay accounting for 36%, 40%, and 23%, respectively.

We used four bays of paddy fields; each bay had a pair of FACE and ambient control plots. The FACE plot was octagonal and 240 m^2^ with an internal diameter of 17 m. CO_2_ emission tubes were installed on eight peripheral sides, and pure CO2 was released from the windward sides to maintain [CO_2_] in the center of the plot at 200 μmol mol^−1^ higher than the ambient control during daylight hours throughout the growing season. The season-long daytime average [CO_2_]±day-to-day standard deviation in ambient [CO_2_] plots was 379±13.9 μmol mol^−1^ and that in elevated [CO_2_] plots was 560±26.3 μmol mol^−1^ in 2011 (36).

Within the FACE and ambient [CO_2_] plots, three kinds of sub-treatments were imposed by separating the plots with PVC corrugated boards (split-plot design with four replicates): an elevated temperature sub-treatment (ET), in which soil and water temperatures for a 3 m×5.4 m area were increased by 2.0°C with heating wires installed on the submerged soil surface between rows; a normal temperature (NT) sub-treatment without a heater; and a no nitrogen sub-treatment (LN) (43), without N fertilizer, but with phosphorous and potassium fertilizers, which were applied before submergence just prior to cultivation at 4.4 g P m^−2^ and 8.3 g K m^−2^, respectively. ET and NT plots both received a standard level of nitrogen just prior to puddling at an amount of 8 g N m^−2^ (2 g m^−2^ N as urea and 6 g as controlled release fertilizer). Soil temperatures in NT and ET during the treatment period averaged 25.2±2.3°C and 27.0±2.5°C, respectively (36).

On 25 April 2011, pre-germinated rice seeds (*Oryza sativa* L. cv. Koshihikari) were sown in seedling trays and placed in the puddled open field. Seedlings at the 5-leaf stage were transplanted on 25 May 2011 by hand at a density of 22.2 hills m^−2^ (a hill is a group of seedlings transplanted to one spot). In the present study, we planted three seedlings per hill at a distance of 15 cm between the plants and 30 cm between the rows. Fields were kept flooded until late August, at which time the surface water was withdrawn for harvesting.

CH_4_ fluxes were measured 13, 27, 41, 48, 62, 69, 76, 83, and 90 d after rice transplanting (DAT) (30). Soil was sampled at the panicle initiation (PI) stage on 5 July (41 DAT) and the mid-ripening (MR) stage on 25 August (92 DAT), 2011. At each sampling time, we dug three blocks of soil at a length of 30 cm, width of 15 cm, and depth of 15 cm, each having one rice hill at its center, from each replicate of each treatment in the paddy fields. Seventy-two soil blocks (3 soil blocks×2 CO_2_ levels×three sub-treatments×4 replicates) were taken and immediately transported to the laboratory at NIAES. Soil samples in the upper (0–1 cm) and lower layers (1–10 cm) were collected from the four angle corners of the three soil blocks in a plot of each treatment and then mixed together at each depth of soil. Fresh soil samples were stored at −20°C until used.

### DNA extraction, PCR amplification, and DGGE analysis

DNA extraction from soil samples was conducted with the Fast DNA SPIN Kit for soil (MP Biomedicals; Solon, OH, USA). PCR amplification was performed using the primer set 1106F-GC/1378R (38) and A189F-GC/Mb661R (26), which targeted the 16S rRNA gene of methanogenic archaea and *pmoA* gene of methane-oxidizing bacteria. PCR programs were detailed in Supplemental Table S1. Approximately 200 ng of PCR products were subjected to the DGGE analysis.

DGGE was performed with a Dcode Universal Mutation Detection System (Bio-Rad Laboratories, Hercules, CA, USA) and the denaturant gradient for gels of methanogenic archaea and methane-oxidizing bacteria ranged between 32 and 62% and between 30 and 70%, respectively, in which 100% denaturant contained 7 M urea and 40% formamide. Electrophoresis was run for 14 h for methanogenic archaea and 16 h for methane-oxidizing bacteria at 60°C and 100 V. Gels were stained for 20 min with the SYBR Green I nucleic acid gel stain (Lonza, Rockland, ME, USA). The stained gel was immediately scanned with an image analyzer (Typhoon 9400, Amersham Bioscience/GE Healthcare UK, Buckinghamshire, UK). Cluster and principal component analyses were performed for the data obtained from the DGGE band patterns based on the intensities of the DGGE bands (0, no band; 1, weak; 2, moderate; 3, strong) using the Black Box Program (1).

### Real-time quantitative PCR

Real-time PCR was used to quantify methanogenic archaeal *mcrA* and methane-oxidizing bacterial *pmoA* genes in soil samples, which encode a key enzyme for methanogenesis (methyl-coenzyme M reductase α subunit) and a 27-kDa polypeptide of the membrane-bound particulate methane monooxygenase, respectively. Real-time PCR was performed using a Thermal Cycler Dice Real Time System (TaKaRa, Otsu, Japan) and the primers mcrA-f and mcrA-r (25) and A189-f and mb661-r (9, 13) were used for quantification. Fragments from methanogenic archaeal or methane-oxidizing bacterial strains were used to make a standard curve for quantification. The standard curve for methanogenic archaea was described previously by Watanabe *et al.* (39). The standard curve for methane-oxidizing bacteria was constructed by amplifying the *pmoA* genes derived from *Methylosinus trichosporium* strain OB3b (U31650) and *Methylomonas koyamae* strain Fw12E-Y (AB538965), and mixing them together to gain a known copy number of the *pmoA* gene for quantification. In both genes, a mixture of a 10-fold dilution series of known numbers covering seven orders of magnitude from 10^1^ to 10^7^ copies of the gene in each assay was used. Real-time PCR was performed in 25 μL of the reaction mixture, which contained 12.5 μL of SYBR Premix Ex Taq, 0.1 μL of the primer set mcrA-f/mcrA-r (50 μM) or A189-f/mb661-r (50 μM) each, 1 μL of template DNA, and 11.3 μL of water. Blanks were always run with sterile water as the template instead of soil DNA. The programs used for the real-time PCR of *mcrA* (22) and *pmoA* (18) genes were shown in Supplemental Table S1. Replicates of each measurement were performed for each extracted DNA sample and the numbers of copies g^−1^ dry soil of methanogenic archaeal and methane-oxidizing bacterial genes were then calculated.

### Statistical analysis

The copy numbers of the *mcrA* and *pmoA* genes in the upper and lower soil layers were logarithmically transformed and subjected to ANOVA, with [CO_2_] (FACE and ambient) as the main plot factor, temperature and nitrogen (NT, ET and LN) as split plot factors, and growth stages (PI and MR) as split-split plot factors. This was followed by the least significant difference (LSD) test to assess significance among the split plot factors. The relationship between methane emission and the ratio of *mcrA*/*pmoA* genes was analyzed by Spearman’s rank correlation method with Ekuseru-Toukei 2012 (Social Survey Research Information, Tokyo, Japan).

## Results

### Composition of methanogenic archaeal and methane-oxidizing bacterial communities

**Fig. 1:**
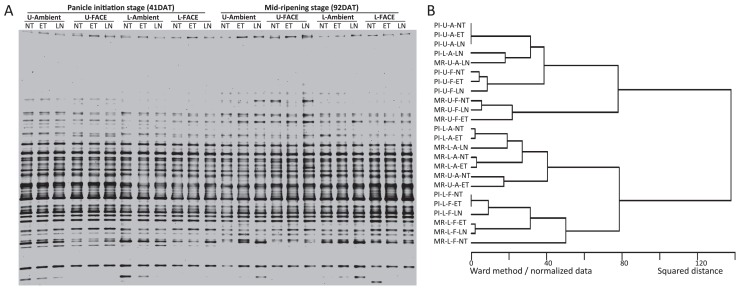
(A) DGGE band patterns of the methanogenic archaeal community from block 3 in ambient and FACE plots at two rice-growing stages in 2011 and (B) a cluster analysis of patterns. PI, panicle initiation stage; MR, mid-ripening stage. U, upper soil (0–1 cm); L, lower soil (1–10 cm). A, ambient; F, FACE. NT, normal temperature; ET, elevated temperature; LN, no nitrogen fertilization.

**Fig. 2:**
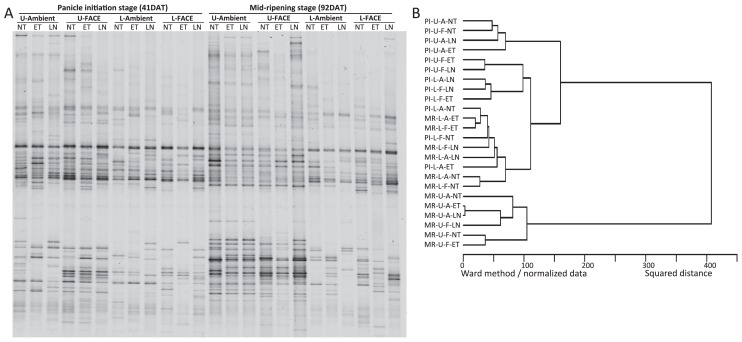
(A) DGGE band patterns of the methane-oxidizing bacterial community from block 3 in ambient and FACE plots at two rice-growing stages in 2011 and (B) a cluster analysis of patterns. PI, panicle initiation stage; MR, mid-ripening stage. U, upper soil (0–1 cm); L, lower soil (1–10 cm). A, ambient; F, FACE. NT, normal temperature; ET, elevated temperature; LN, no nitrogen fertilization.

All samples from ambient and FACE paddy plots, which consisted of the three sub-treatments (NT, ET, and LN) in the upper and lower soil layers at two rice-growing stages in four blocks, were subjected to the DGGE analysis of methanogenic archaea and methane-oxidizing bacteria (Supplemental Fig. S1 and S2). DGGE band patterns with the results of the cluster analysis on the patterns from one of the blocks are shown as representatives (Fig. 1 and 2) because similar results were obtained in four blocks. The DGGE band patterns of the methanogenic archaeal community showed no significant change with elevated [CO_2_] in the paddy fields, and also no significant change by the ET and LN treatments in the ambient and FACE plots (Fig. 1A). A cluster analysis of the band patterns further confirmed that no specific grouping was formed by any treatments, soil layers, or rice-growing stages (Fig. 1B).

**Fig. 3:**
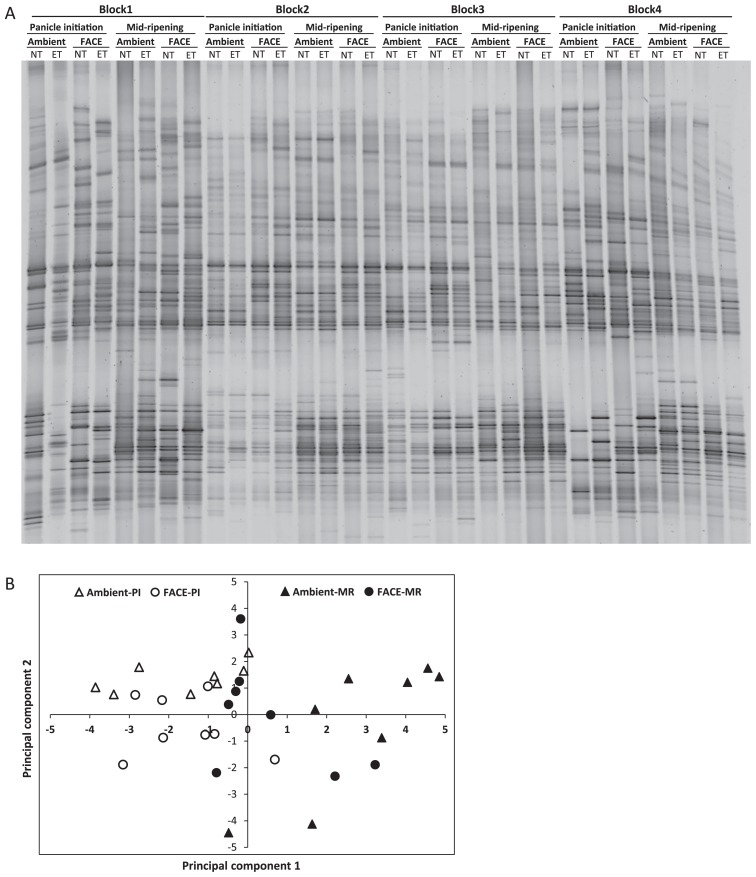
(A) DGGE band patterns of the methane-oxidizing bacterial community from all blocks, except for the LN treatment, in ambient and FACE plots at two rice-growing stages in the upper soil layer in 2011 and (B) a principal component analysis of patterns. NT, normal temperature; ET, elevated temperature.

The DGGE band patterns of the methane-oxidizing bacterial community in block 3 are shown in Fig. 2. A cluster analysis of the DGGE band patterns showed that a separate group was formed by the upper soil layer at the MR stage. The elevated [CO_2_], ET, and LN treatments did not form any specific grouping (Fig. 2B). In the upper soil layer, methane- oxidizing bacterial communities at the PI stage were different from those at the MR stage, but were not in the lower soil layer. Based on these results, a DGGE analysis was performed on methane-oxidizing bacterial communities only in the upper soil layer in all four blocks, except for the LN treatment (Fig. 3A). A principal component analysis (PCA) of the DGGE band patterns indicated that it was possible to separate the methane-oxidizing bacterial community by rice growing stages rather than the elevated [CO_2_] treatment (Fig. 3B).

### Abundance of methanogenic archaea and methane-oxidizing bacteria

**Fig. 4:**
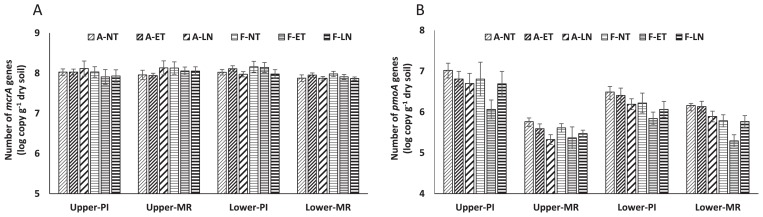
Copy numbers of (A) *mcrA* genes of methanogenic archaea and (B) *pmoA* genes of methane-oxidizing bacteria in ambient and FACE paddy plots at two rice-growing stages in 2011 in upper and lower soil layers. The number stands for log10 values. A, ambient; F, FACE. PI, panicle initiation stage; MR, mid-ripening stage. NT, normal temperature; ET, elevated temperature; LN, no nitrogen fertilization. Error bars represent the standard deviation of the mean values of duplicate measurements from four replicate blocks.

The copy numbers of the *mcrA* gene of methanogenic archaea are shown in Fig. 4A. ANOVA and the LSD test showed that the copy numbers of the *mcrA* gene did not significantly vary with the elevated [CO_2_], ET, and LN treatments or the rice-growing stages in the upper soil layer. In the lower soil layer, the copy numbers of the *mcrA* gene were significantly higher at the PI stage than at the MR stage (*P*<0.01) and were significantly lower with the LN treatment (*P*<0.01) (Table 1). The interaction between the elevated [CO_2_] and LN treatments was significant (*P*<0.05) (Table 1); Fig. 4A indicates that the FACE treatment slightly increased the number of the *mcrA* gene in the NT plot, whereas the LN treatment had no effect.

Unlike the *mcrA* gene, the copy numbers of the *pmoA* gene of methane-oxidizing bacteria clearly varied (Fig. 4B). They were markedly lower at the MR stage than at the PI stage in the upper soil layer. ANOVA and the LSD test showed that the copy numbers of the *pmoA* gene were significantly decreased by the elevated [CO_2_] (*P*<0.05/0.01) and LN treatments (*P*<0.01/0.05) and were significantly lower at the MR stage (*P*<0.01) than at the PI stage in the upper and lower soil layers. The copy numbers of the *pmoA* gene were significantly decreased by the ET treatment (*P*<0.01) in the upper, but not in lower soil layer. The interactions between the elevated [CO_2_] and ET treatments (*P*<0.01) in both soil layers reflected the pronounced reducing effect of elevated [CO_2_] in the ET rather than in the NT treatment (Table 1 and Fig. 4B). The interactions between the elevated [CO_2_] and LN treatments was significant (*P*<0.01) in the lower soil layer (Table 1), and Fig. 4B indicates that the reducing effect of elevated [CO_2_] was more moderate in the LN treatment than in the NT treatment.

### Relationship between methane emission and the mcrA/pmoA ratio

**Fig. 5:**
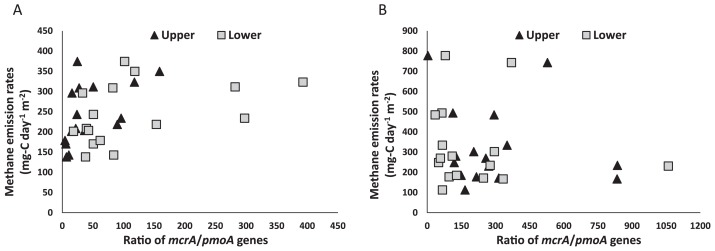
Relationship between the net methane flux from the rice paddy field under two CO_2_ levels combined with NT and ET (30) and the ratio of copy numbers of two key genes, *mcrA*/*pmoA* in upper and lower soil layers at (A) PI and (B) MR rice-growing stages. PI, panicle initiation stage; MR, mid-ripening stage.

The *mcrA*/*pmoA* ratio was calculated in order to evaluate the effects of the elevated [CO_2_] and ET treatments on methane emission in the paddy field (Fig. 5). A positive correlation was observed between methane emission and the ratio of *mcrA*/*pmoA* genes in the upper (*P*<0.01) and lower (*P*<0.05) soil layers at the PI stage by Spearman’s rank analysis.

## Discussion

Elevated [CO_2_] and ET contribute to increases in the total biomass and tiller numbers of rice plants (11, 16, 35, 36) as well as organic substrate input for methanogenesis in the belowground of paddy field soils (4, 24). The present study indicated that neither the methanogenic archaeal community nor its abundance responded to elevated [CO_2_] or ET in the bulk soil of the paddy field, particularly in the upper soil layer. In the field-scale, the results of the present study were consistent with previous findings by Liu *et al.* (23). Previous studies that examined the effects of elevated [CO_2_] and temperature on the viable population of methanogenic archaea also showed positive feedback on methane production with elevated [CO_2_] (10, 16) and soil temperature (10). Although elevated [CO_2_] and ET have the ability to change methane production by increasing labile organic substrate input in the belowground of paddy field soil (40), the increase in organic substrate input may have not been sufficiently large to change the community composition and abundance of methanogenic archaea, and just contributed to higher metabolic activity in response to an increase in methanogenic substrates.

The abundance of methanogenic archaea in lower soil decreased slightly with the LN treatment and at the MR stage. Since exchangeable nitrogen derived from mineralized N or fertilizer N in soil is generally depleted in the late stages of rice growth, N conditions at the MR stage were likely to be poor, even with the NT and ET treatments in which N fertilizer was applied prior to planting, and this may have resulted in a similar reduction in the abundance of methanogenic archaea at the MR stage to that observed with the LN treatment. In addition, the LN treatment with elevated [CO_2_] enhanced decreases in the abundance of methanogenic archaea (Fig. 4). The depletion of available nitrogen under elevated [CO_2_] in soil induced a high C/N ratio and restricted the decomposition of organic matter such as sloughed roots and root exudates, which may have resulted in a decrease in methanogenic substrates (44). Although the effects of limited N conditions on the methanogenic archaeal community structure have not yet been examined, depleted N resources may have slightly reduced the abundance of the methanogenic archaeal community. On the other hand, consistent with previous findings, the methanogenic archaeal community was not sensitive to different cropping systems such as double-cropping (2, 3, 38) or winter-flooding (21), suggesting that it is generally robust against changes in the surrounding environment. Therefore, the present results provide additional evidence for the robust nature of the methanogenic archaeal community.

The methane-oxidizing bacterial community was changed by rice-growing stages in upper soil, but not by the elevated [CO_2_], ET, or LN treatment. In contrast, the abundance of methane-oxidizing bacteria was lower with the elevated [CO_2_], ET, or LN treatment and at the MR stage than with ambient [CO_2_], NT, and at the PI stage. These changes were prominent in the upper soil layer of the paddy field, which partially confirmed previous findings by Yue *et al.* (41).

Lee *et al.* (20) also showed that the methane-oxidizing bacterial community changed between different rice-growing stages in a flooded paddy field in Korea. A parallel study to the present study demonstrated that the abundance of methane-oxidizing bacteria in rice roots decreased under elevated [CO_2_], which interacted with elevated soil temperature in the paddy field (31). The results of the present study demonstrated that a similar effect occurred in bulk soil. The abundance of methane-oxidizing bacteria was previously reported to be decreased by elevated [CO_2_] in meadow soil (19) and in the upper soil layer of a rice-wheat rotated field; the opposite was found in the lower soil layer (41). Moreover, in a soil microcosm experiment, the reduced methane-oxidizing bacterial population reflected decreased methane oxidation under elevated [CO_2_] and temperature conditions (10, 27). These findings support the abundance of the methane-oxidizing bacterial community being decreased by the elevated [CO_2_] and ET treatments in paddy soils.

A possible reason for the decrease in the abundance of methane-oxidizing bacteria may be the depletion of resources, such as nitrogen and oxygen. Exchangeable nitrogen in soil induced by elevated [CO_2_] (44) may have affected methane-oxidizing bacteria, as discussed above for methanogenic archaea; however, the promoting and inhibiting effects of nitrogen fertilization on methane oxidation have been reported (5). Although the structure of soil bacterial communities was not affected by elevated [CO_2_] or ET in the same field as that in the present study (32), the soil microbial biomass was significantly increased by the elevated [CO_2_] treatment (15). This increase in the microbial biomass may have led to competition for O_2_ and induced greater soil respiration under elevated [CO_2_] conditions, resulting in lower O_2_ availability, which, in turn, may have decreased the abundance of methane-oxidizing bacteria. Another reason may be the optimum temperature for the growth of methane-oxidizing bacteria, which generally have mesophilic features (7). Mohanty *et al.* (27) reported that maximum methane consumption by the methane-oxidizing bacterial community occurred between 25 and 35°C and this activity was lost at temperatures greater than 40°C in paddy soil. Even if soil temperatures did not exceed the maximum, the ET treatment in the present study may have affected the abundance of methane-oxidizing bacteria. Enhancements in the decrease observed in the abundance of methane-oxidizing bacteria by the ET treatment with elevated [CO_2_] (Table 1 and Fig. 4) appear to support these findings. Further investigations on the effects of elevated [CO_2_] on the methane-oxidizing bacterial community and its activity are warranted. Although methane emission from paddy fields is mostly through rice plants (29), the significantly decreased abundance of methane-oxidizing bacteria not only in rice roots (30, 31), but also in bulk soil in the present study may have been related to the increased emission of methane from the paddy field under elevated [CO_2_] and ET conditions, as shown by Okubo *et al.* (30).

The ratio of the *mcrA*/*pmoA* genes correlated with methane emission in ambient and FACE paddy plots at the PI stage (Fig. 5A). The copy number of the *pmoA* gene in the upper soil layer correlated with methane emission, whereas no correlation was observed in the lower soil layer at the PI stage (data not shown), indicating the greater contribution of methane-oxidizing bacteria in the upper, than in the lower soil layer to methane emission. Previous studies used the ratio of the *mcrA*/*pmoA* genes (14) or *mcrA*/*pmoA* transcripts (20) as a parameter to assess methane emission in paddy fields. In the present study, although no correlation was found at the MR stage, possibly due to large variations in methane emission rates (Fig. 5B), the ratio of the *mcrA* gene (methanogenic archaea) and *pmoA* gene (methane-oxidizing bacteria) also has potential as an indicator for evaluating methane metabo lism in paddy fields. However, this requires further confirmation in analyses of the mRNAs of the genes of methanogenic archaea and methane-oxidizing bacteria at several rice-growing stages.

In conclusion, the absence of nitrogen fertilization resulted in the low abundance of methanogenic archaea, which were stimulated by elevated [CO_2_]. The abundance of the methane-oxidizing bacterial community in paddy soil was markedly affected by elevated [CO_2_], soil temperature, and the rice-growing stage. The ratio of the *mcrA*/*pmoA* genes correlated with methane emission from the ambient and FACE plots at the PI stage. However, these results need to be investigated further because these responses were based on an analysis at the DNA level. The effects of the elevated [CO_2_], ET, and LN treatments on the active community composition and abundance of methanogenic archaea and methane-oxidizing bacteria currently remain unknown. An analysis at the mRNA level under long-term field conditions at multiple rice-growing stages is necessary in the future in order to broaden our understanding on the responses of the methanogenic archaeal and methane-oxidizing bacterial community compositions and their abundance to the elevated [CO_2_] and temperature treatments.

## Supplementary Information



## Figures and Tables

**Table 1:** Mean value and variance analysis of logarithmically transformed copy numbers of *mcrA* and *pmoA* genes of methanogenic archaea and methane-oxidizing bacteria in ambient and FACE paddy plots.

	Mean				

		*mcrA* gene		*pmoA* gene	
	
		Upper	Lower	Upper	Lower
Whole plot: CO_2_ (a)	A	8.04	7.98	6.58	6.25
	F	8.02	8.02	6.35	5.91
Split plot: NT, ET, and LN (b)	NT	8.04	8.02	6.65	6.23
	ET	7.99	8.04	6.31	6.08
	LN	8.06	7.93	6.42	6.01
Split-split plot: stage (c)	PI	8.01	8.07	6.76	6.25
	MR	8.05	7.91	5.55	5.92

Analysis of variance					

Factor	d*f*	*mcrA* gene		*pmoA* gene	
	
		Upper	Lower	Upper	Lower

Replicate	3				
Whole plot: CO_2_ (a)	1	ns	ns	^*^	^**^

Main plot error	3				
Split plot: NT, ET, and LN (b)	2	ns	^**^	^**^	^*^
Temperature: ET vs NT (b1)		ns	ns	^**^	ns
N: LN vs NT (b2)			^**^	^**^	^*^
a×b	2	ns	^*^	^**^	^**^
a×b1			ns	^**^	^**^
a×b2			^*^	ns	^**^
Split-split plot: stage (c)	1	ns	^**^	^**^	^**^
a×c	1	ns	ns	ns	ns
b×c	2	ns	ns	ns	ns
a×b×c	2	ns	ns	ns	ns
Split-split error	18				

A, ambient; F, FACE. PI, panicle initiation stage; MR, mid-ripening stage. NT, normal temperature; ET, elevated temperature; LN, no nitrogen fertilization.

** and * show a significant difference at *P*<0.01 and *P*<0.05, respectively, from the control treatment (ambient for factor a, normal temperature for factor b, and panicle initiation stage for factor c) or for interactions between factors based on a variance analysis and the LSD test for a split-split plot-arranged experiment.

ns shows no significant difference.
