# Supplementary material for: Effect of Elevated CO2 Concentration, Elevated Temperature and No Nitrogen Fertilization on Methanogenic Archaeal and Methane-Oxidizing Bacterial Community Structures in Paddy Soil
Source: Microbes Environ. 2016 Sep 7;31(3):349–56. doi: 10.1264/jsme2.ME16066 (PMC5017813; doi:10.1264/jsme2.ME16066)
Supplement: Supplementary file 1 [file 31_349_s1.pdf]

Supplemental Fig. S1 DGGE band patterns of the methanogenic archaeal community from blocks 1, 2, and 4 in ambient and FACE plots at two rice-growing stages in 2011. U, upper soil (0-1 cm); L, lower soil (1-10 cm). A, ambient; F, FACE. NT, normal temperature; ET, elevated temperature; LN, no nitrogen fertilization.

Supplemental Fig. S2 DGGE band patterns of the methane-oxidizing bacterial community from blocks 1, 2, and 4 in ambient and FACE plots at two rice-growing stages in 2011. U, upper soil (0-1 cm); L, lower soil (1-10 cm). NT, normal temperature; ET, elevated temperature; LN, no nitrogen fertilization.

Supplemental Table S1 PCR and real-time quantitative PCR programs of methanogenic archaea and methane-oxidizing bacteria.

	**primer set**	**initial denaturation**	**cycles**	**denaturation**	**annealing**	**extension**
PCR	1106F-GC/1378R	94℃ 30s	35	95℃ 30s	55℃ 30s	72℃ 90s
	A189f-GC/mb661r	94℃ 30s	35	95℃ 30s	56℃ 30s	72℃ 60s
Real-time PCR	mcrA-f/mcrA-r	95℃ 30s	45	95℃ 40s	55℃ 40s	72℃ 30s
	A189f-GC/mb661r	95℃ 30s	45	95℃ 30s	65.5℃ 30s	72℃ 45s

Supplemental Fig. S1

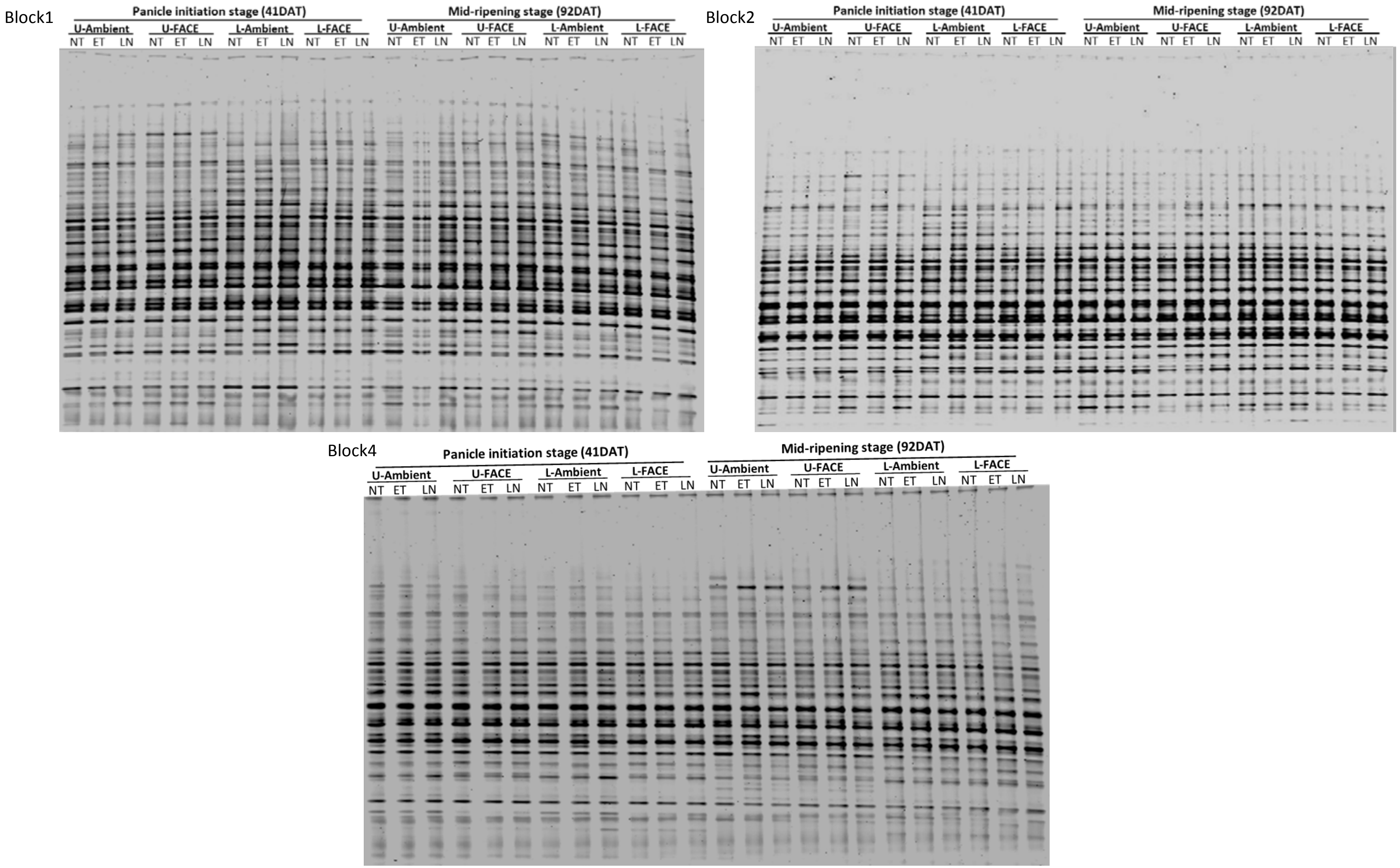

Supplemental Fig. S1 DGGE band patterns of methanogenic archaeal community from the blocks 1, 2 and 4 in ambient and FACE plots at two rice growing stages in 2011. U, upper soil (0-1 cm); L, lower soil (1-10 cm). NT, normal temperature; ET, elevated temperature; LN, no nitrogen fertilization.

Dongyan Liu

Supplemental Fig. S2

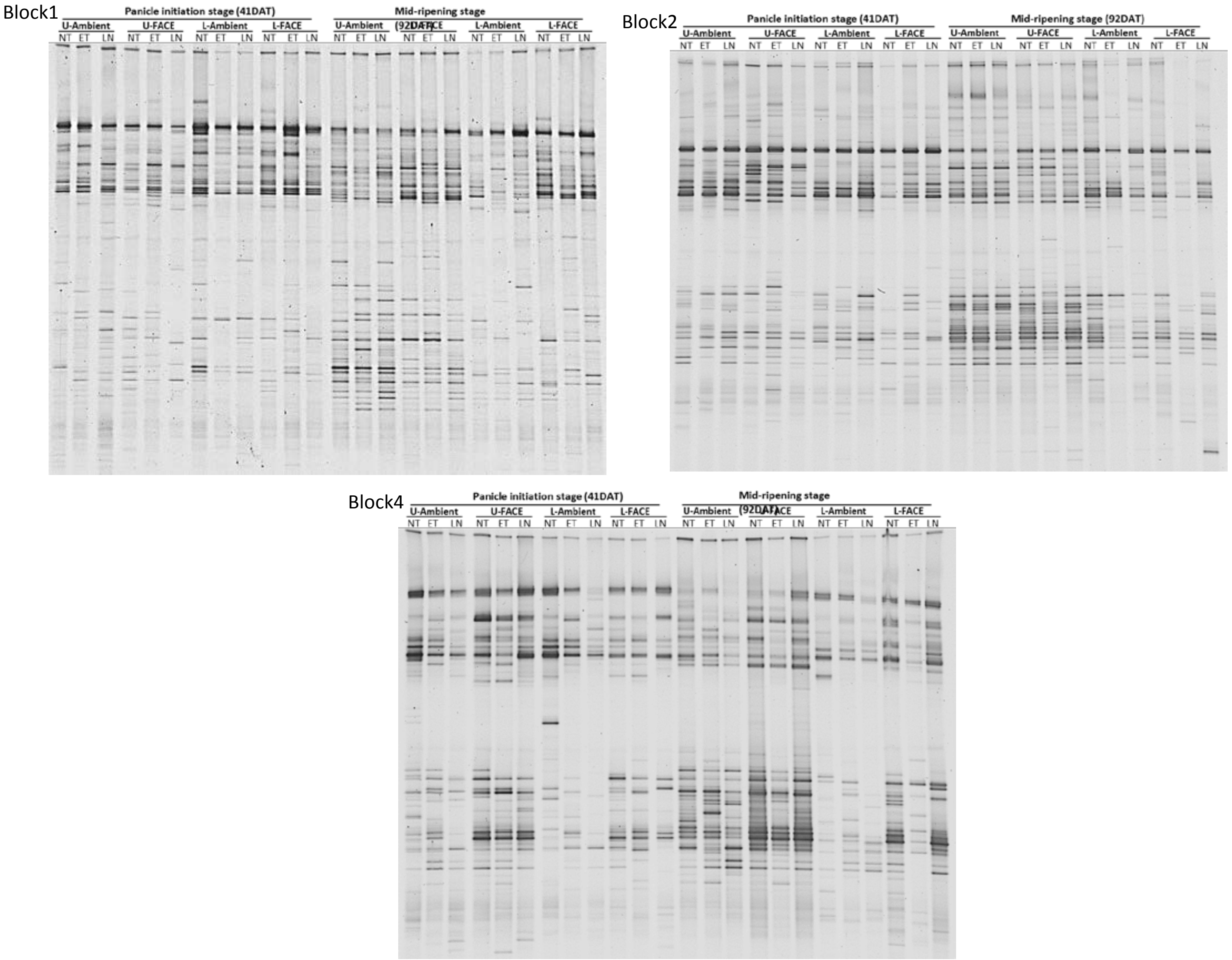

Supplemental Fig. S2 DGGE band patterns of methane-oxidizing bacterial community from the blocks 1, 2 and 4 in ambient and FACE plots at two rice growing stages in 2011. U, upper soil (0-1 cm); L, lower soil (1-10 cm). NT, normal temperature; ET, elevated temperature; LN, no nitrogen fertilization.

Dongyan Liu
